# The Use of Bispectral Index Monitoring Does Not Change Intraoperative Exposure to Volatile Anesthetics in Children

**DOI:** 10.3390/jcm9082437

**Published:** 2020-07-30

**Authors:** Cornelius A. Sullivan, Chinyere Egbuta, Raymond S. Park, Karina Lukovits, David Cavanaugh, Keira P. Mason

**Affiliations:** 1 Department of Anesthesiology, Critical Care and Pain Medicine, Boston Children’s Hospital, Harvard Medical School, Boston, MA 02115, USA; Cornelius.Sullivan@childrens.harvard.edu; 2 Department of Anesthesiology, Critical Care and Pain Medicine, Boston Children’s Hospital, Boston, MA 02115, USA; Chinyere.Egbuta@childrens.harvard.edu (C.E.); Raymond.Park@childrens.harvard.edu (R.S.P.); Karina.Lukovits@childrens.harvard.edu (K.L.); 3 Boston Biostatistical Consulting, North Reading, MA 01864, USA; dmcav6@gmail.com

**Keywords:** BIS, pediatrics, volatile anesthetic, neurocognitive dysfunction, anesthesia

## Abstract

The exposure of infants and children to volatile anesthetics, such as sevoflurane, has been a topic of concern with respect to the potential risk for long term neurocognitive effects. The primary objective of this study was to determine whether the perioperative utilization of Bispectral Index (BIS) monitoring alters the sevoflurane delivery and exposure to children. This is a prospective randomized trial of two groups of healthy ambulatory day surgery patients (2 to 12 years). The patients in both groups had the BIS applied soon after the induction of general anesthesia, but only the anesthesiologists in the group randomized to BIS visible were able to see the BIS values. All of the patients received general anesthesia with sevoflurane. This study found no difference in the overall exposure to sevoflurane between both groups (mean end-tidal sevoflurane level of 1.8 in both groups, *P* = 084). The duration of time in the recovery room, the time to meet discharge criteria, the Pediatric Agitation Emergence Delirium (PAED) scores and the Face, Legs, Activity, Cry, Consolability (FLACC) scores were not statistically different between the groups. The application and utilization of intraoperative BIS monitoring does not alter the sevoflurane administration nor the discharge readiness nor the recovery profile in healthy ambulatory children.

## 1. Introduction

There is considerable interest and some concern over the potential risk of general anesthesia, volatile anesthetics in particular, on the developing brain [1,2,3,4]. This potential for neurocognitive effects led the Food and Drug Administration (FDA) to release a Safety Statement in 2017, approving label changes for general anesthetics in young children. The FDA advised that consideration be given to delaying potentially elective surgery in young children when medically appropriate [5]. Limiting a child’s exposure to volatile general anesthetics could have important value, particularly with the ongoing concern for long-term neurocognitive dysfunction from volatile anesthetics. The Bispectral Index Monitor (BIS) is a non-invasive monitor that translates electroencephalographic data from an adhesive applied to the forehead to numerical values. These values are meant to be indicative of the depth of anesthesia. Although commonly used, the BIS has not been adopted as a standard of care for pediatric anesthesia. Various studies have been conducted to assess whether BIS monitoring can offer a reliable, objective evaluation of anesthetic depth in children, but to date, the results are inconclusive [6,7,8,9].

With the heightened sensitivity to the potential neurocognitive effects of volatile anesthetics, such as sevoflurane, in the pediatric population, it would be especially valuable to determine whether BIS monitoring could alter the amount of sevoflurane delivered. The primary objective of this study was to determine whether access to perioperative BIS monitoring alters the amount of sevoflurane delivered. The hypothesis was that BIS monitoring would decrease the amount of volatile anesthetic delivered. The secondary outcomes were to determine whether BIS monitoring alters pediatric post-operative recovery profiles. The most important conclusion of this study was that BIS monitoring did not significantly alter the amount of volatile anesthetic, and, for now, should not be considered as a useful monitor to decrease exposure to volatile anesthetics in the pediatric population.

## 2. Materials and Methods

**Figure 1:**
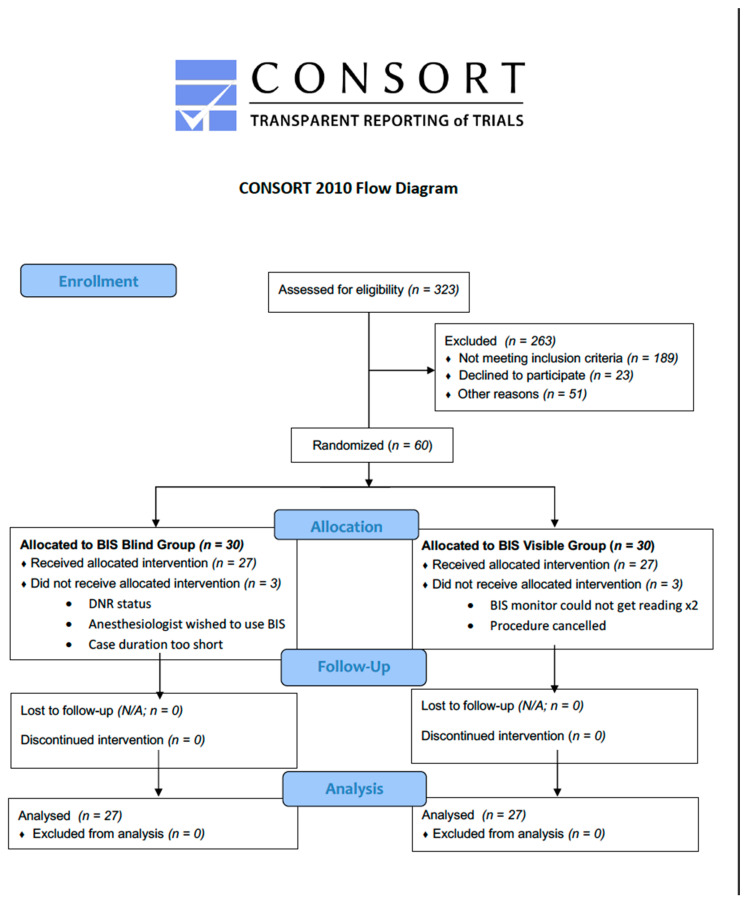
CONSORT 2010 Flow Diagram.

This is a prospective randomized parallel arm clinical trial of ambulatory (ASA 1 and ASA 2) day surgery patients (2–12 years) presenting for orthopedic procedures (NCT03052543) (Consort Diagram, Figure 1). All patients gave their informed consent for inclusion before they participated in this study. The study was conducted in accordance with the Declaration of Helsinki, and ethical approval for this study (IRBP00024412) was provided by the Boston Children’s Hospital Internal Ethics and Review Board on 29 December 2016. The patients were randomized into 2 groups, identified as BIS blinded (test) and BIS visible (control), of 30 patients each. The randomization was generated using a blocking randomization scheme, allocating subjects in a 1:1 even allocation to the two groups. The randomization was performed by the statistician using SAS v9.4. All had the BIS sensor placed and recorded as soon after the induction as the patient was able to tolerate placement. The BIS blinded group had the BIS monitor and numerical data physically hidden from view, unable to be visualized by the anesthesiologist. The BIS visible group had the BIS monitor and numerical data visible to the anesthesiologist. The BIS sensor remained adhered on the forehead, recording data, until the completion of the general anesthetic. The anesthesiologist designated, “Move Patient to Post Op” on the Electronic Medical Record as an indicator that the anesthetic had been completed, the BIS sensor had been removed and that the patient was ready for transport from the operating room to the post-anesthesia care unit (PACU). The anesthesia providers of both groups of patients did not receive any directive as to the anesthetic delivery technique or agents. All the patients received a general anesthetic with a volatile agent. The anesthesiologists randomized to the BIS visible group had the numerical values visible and were able to use these values to alter or direct the anesthetic delivery. All anesthesiologists were experienced in the interpretation and usage of BIS monitoring. In general, BIS values of 40–60 are indicative of a state of general anesthesia [10].

The absolute exclusion criteria includes anesthesiologist refusal to participate in the study, patient/parent refusal, allergy to the BIS adhesive, neurologic impairment, and a history of seizures (Table 1). The primary outcome of this study compares the intraoperative numerical BIS values and the mean end-tidal Sevoflurane values in the BIS visible and the BIS blinded groups. The secondary outcomes compare the recovery parameters between the groups: the duration of time in recovery, the time to meet an Aldrete score of 9, the Pediatric Anesthesia Emergence Delirium (PAED) scores, the Face, Legs, Activity, Cry, Consolability (FLACC) scores, the Numerical Rating pain score (NRS) and the Wong generalized pain score.

### Statistical Analysis 

The baseline characteristics: gender, age, and weight, were summarized for the BIS visible, the BIS blind, and the overall groups. Normality assumptions were met by use of the Shapiro–Wilk test. The following endpoints were analyzed using descriptive statistics and a *t*-test of mean comparison between groups: the BIS score, the end-tidal sevoflurane concentration, the duration of time in recovery, the duration of anesthesia, the time to an Aldrete score of 9, the agitation/delirium scores, and the pain assessment scores (FLACC, NRS generalized, and Wong generalized). The tests for normality were satisfied using the Shapiro–Wilk test. The area under the curve (AUC) was analyzed for the BIS score and end-tidal sevoflurane. The FLACC categories were summarized by frequency. The frequency of administration types, as well as the type of induction, was summarized. Unless specified, two-sided *p*-values are used. Two-sided values of p < 0.05 will be considered statistically significant. Statistical analyses were performed using SAS^®^ statistical software (version 9.4, SAS^®^ Institute, Cary, NC, USA).

## 3. Results

### 3.1. Baseline Characteristics

The baseline characteristics descriptive statistics can be found in Table 2 and Table 3. Female subjects comprised 74.1% of the BIS visible group, 44.4% of the BIS blinded group, and 59.3% overall. The age range was similar amongst the different groups, with a mean age of 9.0 (SD 3.42) years in the BIS visible group, 9.9 (SD 3.01) years in the BIS blinded group, and 9.5 (SD 3.23) years overall. The summary of endpoints can be found in Table 4. The mean BIS score in the BIS visible group was 43.7 (SD 7.59), and 42.2 (SD 7.84) in the BIS blinded group, with a *p*-value of 0.4935, indicating no significant difference between the groups. Furthermore, the consumption of anesthetic agents may change according to age, so the BIS score by age group [2–6], [7–13] was summarized and is shown in Table 5. The area under the curve was compared between the two groups, with the BIS visible group having a mean AUC of 6079.5 (SD 4989.49), and the BIS blind group having a mean AUC of 3556.6 (SD 1554.43). This resulted in a statistically significant difference, *p*-value 0.0248.

### 3.2. Intra-Operative Management

The end-tidal sevoflurane concentration had no significant difference between the groups, *p*-value 0.8365. Similar to the BIS score, the sevoflurane concentration by age group [2–6], [7–13] was summarized and is shown in Table 5. The area under the curve was compared between the two groups, with the BIS visible group having a mean AUC of 176.4 (SD 87.11), and the BIS blind group having a mean AUC of 195.2 (SD 139.52). This result is not a statistically significant difference, *p*-value 0.5563.

The duration of anesthesia (minutes) is defined as the time the BIS sensor was attached until the time the BIS sensor was removed. The BIS blinded group did have a longer time of anesthesia with a mean anesthesia time of 96.3 (SD 44.08) minutes, while the BIS visible group recovered in 86.9 (SD 36.52) minutes. This difference was not statistically significant, *p*-value 0.3952. 

Propofol was administered in 48 out of the 54 subjects (88.9%), with two subjects from the BIS blind group and four subjects from the BIS visible group not receiving propofol. There was no statistical significance in the propofol administration between the groups, *p*-value 0.6687. Nitrous oxide was administered to 46 out of the 54 subjects (85.2%), with six subjects from the BIS blind group and two subjects from the BIS visible group not receiving nitrous oxide. There was no statistical significance in the nitrous oxide administration between the groups, *p*-value 0.1415. All subjects received Sevoflurane, and no subjects received Ketamine.

The type of anesthesia induction was summarized for both groups, with 28 (51.9%) subjects receiving IV induction and 26 (48.1%) subjects receiving inhalation induction. There was a statistically significant difference (*p*-value 0.0065) in induction methods between the two groups: the BIS blind group had 19 subjects with IV and 8 subjects with inhalation, while the BIS visible group had 9 subjects with IV and 18 subjects with inhalation.

### 3.3. Recovery Profiles

The duration of time in recovery (minutes) is defined as the time from when the BIS was removed until the time the patient was ready to discharge. The BIS visible group did have a longer time in recovery, with a mean recovery time of 54.1 (SD 43.36) minutes, while the BIS blinded group recovered in 43.3 (SD 33.97) minutes. This difference was not statistically significant, *p*-value 0.3174. Only four subjects reached an Aldrete score of 9: three subjects from the BIS visible group, and one subject from the BIS blinded group. In the BIS visible group, the mean time to an Aldrete score of 9 was 119.7 (SD 37.23) minutes, and in the BIS blinded group it was 56.0 (SD NC) minutes. The difference was not statistically significant, *p*-value 0.2768. The BIS visible group had a PAED score of 5.3 (SD 5.02), and the BIS blinded group a score of 3.3 (SD 6.13), which was not a statistically significant difference, *p*-value 0.4280. The FLACC, NRS generalized pain and Wong generalized pain scores were all higher for the BIS blinded group, but no differences between the groups for these scores were statistically significant.

## 4. Discussion

Although widely utilized, the use of intraoperative BIS monitoring for pediatric anesthesia is not a standard of care and is largely based on clinician preference. To date, there is no consensus on the value of the BIS from studies which have examined its usefulness in the pediatric population. In children, it has been shown that there is greater inter-individual variation in the BIS values and the end-tidal sevoflurane correlation compared to adults [7,9], with significant variability depending on age [6]. Some authors cautioned that the BIS may not be a reliable monitor of anesthetic depth for the pediatric population [8]. Other studies, however, reported that BIS monitoring can be associated with reduced end-tidal sevoflurane concentration [11,12] and faster recovery times [11,13]. Using the BIS as a monitor of the depth of anesthesia has been examined as a means to decrease inadvertent intraoperative awareness. Accidental awareness under general anesthesia is estimated to occur in 0.2% of adults, but has been suggested to occur at frequencies of up to 0.8% in children [14,15,16].

In 2017, the FDA released a safety announcement warning that exposure to general anesthetics for “lengthy periods of time or over multiple surgeries or procedures may negatively affect brain development in children younger than 3 years.” The FDA advised that “consideration should be given to delaying potentially elective surgery in young children where medically appropriate” [5]. Since 2017, the anesthesia community, along with the general medical community and the parents of children undergoing anesthesia, have a heightened awareness and sensitivity to the potential risks of volatile anesthetics [2,3,4]. Although to date, anesthetics of short duration have not been shown to affect neurocognitive development in infants for up to 5 years post-exposure, the FDA warning remains [17,18,19,20].

If BIS monitoring can aid in assessing anesthetic depth, reliably improve the perioperative outcomes and, most importantly, decrease the exposure to volatile anesthetics, such as sevoflurane, then it would be a valuable addition to standard of care practices [21,22]. Our study does not support a role for BIS monitoring as a means to decrease exposure to volatile anesthetics in the potentially vulnerable pediatric population. Even without the visible BIS numerical values, anesthesiologists were able to consistently gauge the depth of anesthesia and maintain similar BIS values and volatile anesthetic concentrations between the two groups. There was also no difference in the post-operative recovery parameters. This finding is consistent with many large scale adult studies that have demonstrated that BIS guidance does not alter anesthetic administration on average [23,24], does not lead to a faster recovery [25,26,27], and does not reduce the risk of mortality [28].

There are some limitations of this study. As this study was novel, we were unable to predict a pre-specified effect size. Thus, there is the possibility, although our findings were of statistical significance, that we may be underpowered for detecting any hypothetical effect sizes of interest. All of the anesthesiologists directly involved in this study were experienced pediatric anesthesiologists, with knowledge and expertise on BIS monitoring. That the mean end-tidal sevoflurane concentrations did not differ between the groups, could reflect the clinical experience of the anesthesiologists, and their ability to titrate volatile anesthetic delivery without the aid of a BIS monitor. This experience of the anesthesiologists may have equally supported our findings that there was no difference in the mean intraoperative BIS scores between the groups. The anesthetic agents used did not differ between the groups. Although there was a difference in the propensity for intravenous versus inhalation induction between the two groups, the overall intention of the study, to determine whether the visibility of the BIS values altered the intraoperative BIS values and the delivery of sevoflurane anesthetic, should remain unaffected. Regardless of the induction technique used (intravenous versus inhalation), the intraoperative delivery of inhalation anesthesia and the maintenance of the BIS values remained unchanged between the groups. Future studies are warranted to determine whether less experienced anesthesiologists would manifest similar outcomes. Additionally, this study did not have a pre-specified effect size, so we acknowledge that we may be underpowered for detecting any hypothetic effect sizes of interest.

This is the first study, to our knowledge, that compared the mean amount of volatile anesthetic received as well as the BIS and the recovery outcomes between groups of patients who received anesthetic care with and without the visibility of BIS numerical values. To date, BIS monitoring is not a standard of care in pediatric anesthesia. The concern for limiting the amount of volatile anesthetic exposure to the pediatric population, supports the quest to examine whether BIS monitoring could add a precision to anesthetic delivery that results in lower levels of volatile anesthetic exposure. Our study did not show such an advantage to the BIS. Future studies are necessary to determine whether clinician experience alters the outcome, and, more importantly, whether those with less pediatric experience could benefit and show demonstrable differences in volatile anesthetic delivery in the presence of the BIS.

## Figures and Tables

**Table 1:** Detailed reasons (number of patients) for meeting exclusion criteria.

**Prone Position** **(Contraindicates BIS Application)**	**36**
Planned procedure length too short	25
Research staff unavailable	19
Extended recovery time required	18
History of seizures	15
Developmental delay- unable to obtain assent	10
Adhesive allergy (concern with BIS adhesive)	1
Other (scheduling issues, anesthesia staff or family decline participation, behavioral concerns regarding patient)	65
Total number that were screened and excluded	189

**Table 2:** Baseline characteristics.

	Statistic	BIS Visible (*N* = 27)	BIS Blind (*N* = 27)	Overall (*N* = 54)
Gender				
Female	*n* (%)	20 (74.1)	12 (44.4)	32 (59.3)
Male		7 (25.9)	15 (55.6)	22 (40.7)
Age (years)	*n*	27	27	54
	Mean (SD)	9.0 (3.42)	9.9 (3.01)	9.5 (3.23)
	Median	11.0	11.0	11.0
	Min, Max	2.0, 12.0	2.0, 13.0	2.0, 13.0
Weight (kg)	*n*	27	27	54
	Mean (SD)	36.9 (18.54)	45.9 (18.33)	41.4 (18.82)
	Median	34.4	47.4	40.1
	Min, Max	14.2, 93.1	13.4, 82.1	13.4, 93.1
Height (cm)	*n*	27	27	54
	Mean (SD)	134.5 (24.56)	144.0 (19.97)	139.3 (22.68)
	Median	139.0	148.6	143.0
	Min, Max	91.5, 169.0	90.3, 170.0	90.3, 170.0

**Table 3:** Procedures for Bispectral Index (BIS) Blind and BIS Visible Groups

BIS Blind	BIS Visible
Ankle kidner Hand reconstruction Bone biopsy Exostosis excision Ganglion wrist excision Arthroscopy knee ACL reconstruction with IT band Hardware removal femur proximal unilateral Hardware removal tibia/fibula Bone biopsy/curettage/packing Tendon transfer ankle/foot; tendon lengthening/release achilles Knee arthroscopy with/or without synovial biopsy Physeal arrest lower extremity Right radial head non union Osteotomy toe Tendon lengthening/release achilles Orif radius and/or ulna Epiphysiodesis Cast spica application/change and hip arthrogram Navicular accessory excision Arthroscopy, knee Tendon transfer ankle/foot + Tenotomy achilles, percutaneous Soft tissue benign tumor excision, simple Bone biopsy/curettage/packing, simple; cast application/change Hardware removal femur proximal unilateral complex Arthroscopy, wrist	Trigger thumb and or finger release Exostosis excision Navicular accessory excision Bone biopsy/curettage/packing Bone cyst curettage/packing Tendon lengthening release, unilateral Arthroscopy knee and epiphysiodesis Foot polydactyly excision Arthroscopy knee with MPFL repair Hardware removal buried pin, plate, or screw; arthrogram hip Hand exploration Arthroscopy elbow with arthrotomy Ankle crisman snook Epiphysiodesis Tendon lengthening/release achilles Calcanea navicular coalition excision Bone biopsy/curettage, simple Osteotomy toe Hardware removal buried pin, plate or screw Arthroscopy knee with open medial plication Tenotomy, foot Tendon transfer ankle/foot

**Table 4:** Endpoints.

	Statistic	BIS Visible (*N* = 27)	BIS Blind (*N* = 27)	Overall (*N* = 54)
**BIS Score**	*n*	27	25	52
	Mean (SD)	43.7 (7.59)	42.2 (7.84)	43.0 (7.67)
	Median	43.8	41.5	43.1
	Min, Max	26.9, 62.7	26.4, 55.8	26.4, 62.7
	*p*-value *			0.4935
**End-tidal Sevoflurane Concentration (%)**	*n*	27	27	54
	Mean (SD)	1.8 (0.67)	1.8 (0.64)	1.8 (0.65)
	Median	2.0	1.9	1.9
	Min, Max	0.3, 2.9	0.1, 3.0	0.1, 3.0
	*p*-value *			0.8365
**Duration of time in recovery (mins) [1]**	*n*	26	27	53
	Mean (SD)	54.1 (43.36)	43.3 (33.97)	48.6 (38.87)
	Median	46.0	39.0	40.0
	Min, Max	7.0, 222.0	−37.0 ^, 154.0	−37.0 ^, 222.0
	*p*-value *			0.3174
**Duration of anesthesia (mins) [2]**	*n*	27	27	54
	Mean (SD)	86.9 (36.52)	96.3 (44.08)	91.6 (40.38)
	Median	81.0	81.0	81.0
	Min, Max	35.0, 192.0	45.0, 208.0	35.0, 208.0
	*p*-value *			0.3952
**Time to Aldrete score of 9 (mins)**	*n*	3	1	4
	Mean (SD)	119.7 (37.23)	56.0 (NC)	103.8 (44.02)
	Median	105.0	56.0	98.5
	Min, Max	92.0, 162.0	56.0, 56.0	56.0, 162.0
	*p*-value *			0.2768
**PAED Score**	*n*	11	10	21
	Mean (SD)	5.3 (5.02)	3.3 (6.13)	4.3 (5.53)
	Median	5.0	1.0	3.0
	Min, Max	0.0, 12.0	0.0, 20.0	0, 20.0
	*p*-value *			0.4280
**FLACC Pain Assessment Score**	*n*	26	8	34
	Mean (SD)	0.8 (1.54)	1.4 (2.88)	1.0 (1.90)
	Median	0.0	0.0	0.0
	Min, Max	0, 5	0, 8	0, 8
	*p*-value *			0.6310
**NRS Generalized Pain Score**	*n*	15	20	35
	Mean (SD)	2.6 (2.90)	3.6 (2.30)	3.2 (2.62)
	Median	2.0	4.0	3.0
	Min, Max	0, 8	0, 8	0, 8
	*p*-value *			0.0867
**Wong Generalized Pain Score**	*n*	11	9	20
	Mean (SD)	3.3 (2.57)	3.8 (2.73)	3.5 (2.59)
	Median	2.0	4.0	4.0
	Min, Max	0, 8	0, 8	0, 8
	*p*-value *			0.6758
**Activity—FLACC**	*n* (%)			
Lying quietly, normal position, moves easily (0)		24 (77.4)	7 (22.6)	31 (91.2)
Squirming, shifting back and forth, tense (1)		1 (33.3)	2 (66.7)	3 (8.8)
**Consolability—FLACC**	*n* (%)			
Content, relaxed (0)		21 (77.8)	6 (22.2)	27 (79.4)
Difficult to console or comfort (2)		0	1 (100.0)	1 (2.9)
Reassured by occasional touching, hugging, distractible (1)		5 (83.3)	1 (16.7)	6 (17.7)
**Cry—FLACC**	*n* (%)			
Crying steadily, screams or sobs, frequent complaints (2)		0	1 (100.0)	1 (2.9)
Moans or whimpers; occasional complaint (1)		5 (16.7)	1 (83.3)	6 (17.7)
No cry (awake or asleep) (0)		21 (77.8)	6 (22.2)	27 (79.4)
**Facial—FLACC**	*n* (%)			
Frequent to constant quivering chin, clenched jaw (2)		0	1 (100.0)	1 (2.9)
No particular expression or smile (0)		19 (76.0)	6 (24.0)	25 (73.5)
Occasional grimace or frown, withdrawn, disinterested (1)		7 (87.5)	1 (12.5)	8 (23.5)
**Legs—FLACC**	*n* (%)			
Normal position or relaxed (0)		23 (76.7)	7 (23.3)	30 (88.2)
Uneasy, restless, tense (1)		3 (75.0)	1 (25.5)	4 (11.8)

PAED: Pediatric Anesthesia Emergence Delirium scale; FLACC: Face, Legs, Activity, Cry, Consolability) scale; NRS: Numerical Rating pain score. [1] Duration of time in recovery is calculated as time ready to discharge–time at BIS removed. [2] Duration of anesthesia is calculated as time of BIS sensor off—BIS sensor on. * *p*-value calculated using *t*-test. ^ One subject had a ready for discharge time that is before the time of BIS sensor off.

**Table 5:** BIS Score and end-tidal sevoflurane concentration by age group.

Parameter Statistic	BIS Visible (*N* = 27)		BIS Blind (*N* = 27)	
	Age (2–6)	Age (7–13)	Age (2–6)	Age (7–13)
BIS Score				
*n*	6	21	4	21
Mean (SD)	42.9 (6.44)	43.9 (8.02)	39.5 (10.67)	42.7 (7.41)
Median	41.1	44.5	39.7	41.5
Min, Max	34.9, 53.1	26.95, 62.72	26.4, 52.2	31.3, 55.8
End-tidal Sevoflurane Concentration				
*n*	6	21	4	23
Mean (SD)	2.1 (0.65)	1.7 (0.67)	2.4 (0.47)	1.7 (0.61)
Median	2.2	2.0	2.3	1.7
Min, Max	1.3, 2.9	0.3, 2.7	1.9, 3.0	0.1, 2.6
